# Images in Anesthesiology: Carotid Sinus Reflex From Neck Mass and Opioids

**DOI:** 10.7759/cureus.62048

**Published:** 2024-06-10

**Authors:** Andrea N Vrionis, Kevin R Olsen

**Affiliations:** 1 Morsani College of Medicine, University of South Florida, Tampa, USA; 2 Department of Anesthesiology, Moffitt Cancer Center, Tampa, USA

**Keywords:** head and neck tumors, carotid sinus, opioid, anesthesia, otolaryngology

## Abstract

Surgery on head and neck masses presents unique challenges to overcome, especially in relation to preoperative anesthesia induction. Tumor proximity to the carotid sinus can result in extreme hemodynamic depression, by way of compression or direct invasion of the node. Neck hyperextension required for endotracheal intubation can worsen the underlying compression. Additionally, many anesthetic agents have sympatholytic properties that can exacerbate this imbalance further toward the parasympathetic response. We present a case of a patient with non-Hodgkin lymphoma whose tumor compression of the carotid sinus precipitated an exaggerated vagal reflex response following fentanyl administration.

## Introduction

The carotid sinus is a baroreceptor of the autonomic nervous system that responds to pressure on the arterial wall, eliciting a parasympathetic effect of bradycardia and hypotension. Head and neck tumors lying near the carotid sinus can induce an abnormally strong efferent vagal discharge either via pressure from the mass or through direct invasion of the node [1]. This signal can precipitate extreme sinus bradycardia, hypotension, sinus arrest, or impaired conduction through the atrioventricular (AV) node. This phenomenon, termed carotid sinus syndrome, is particularly likely for tumors located in the parapharyngeal space. Indeed, this intense carotid sinus reflex has previously been noted in otolaryngology surgeries, but less reported is the compounding effect of opioids with preexisting hypersensitivity [2].

## Case presentation

**Figure 1:**
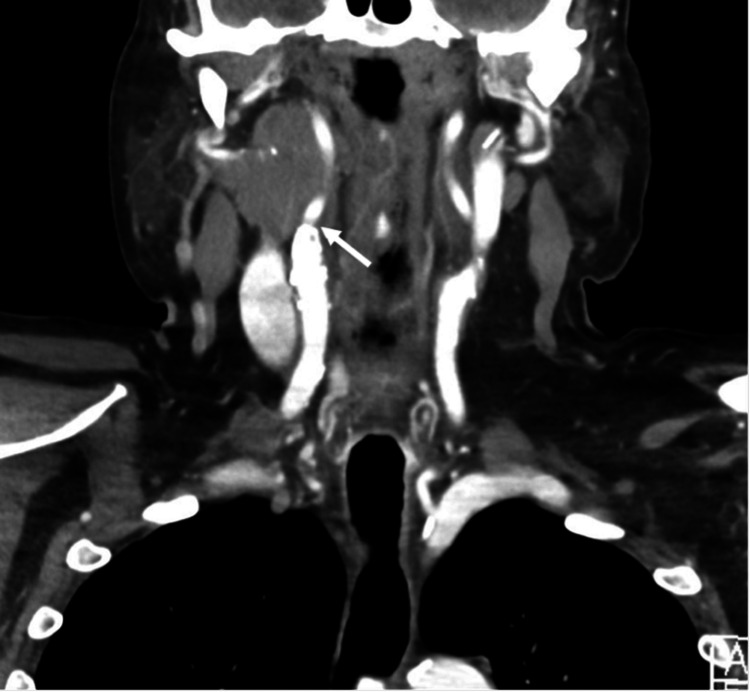
Coronal plane. Computed tomography (CT) of the head and neck reveals a large, level II nodal mass extending from the level of the angle of the mandible superiorly, almost to the skull base in the right parapharyngeal space inferiorly. The arrow points to the bifurcation of the right common carotid artery, illustrating the proximity of the tumor to the carotid sinus.

**Figure 2:**
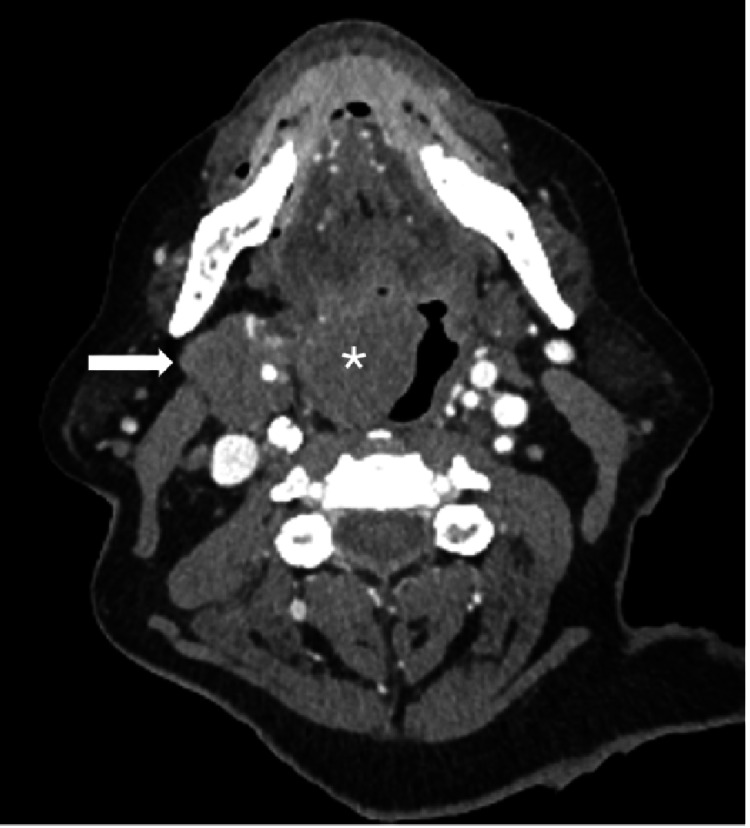
The axial plane offers additional views of the parapharyngeal mass (arrow), as well as the second mass (asterisk) involving the right tongue base with extension to the right tonsil, filling >50% of the oropharynx.

**Figure 3:**
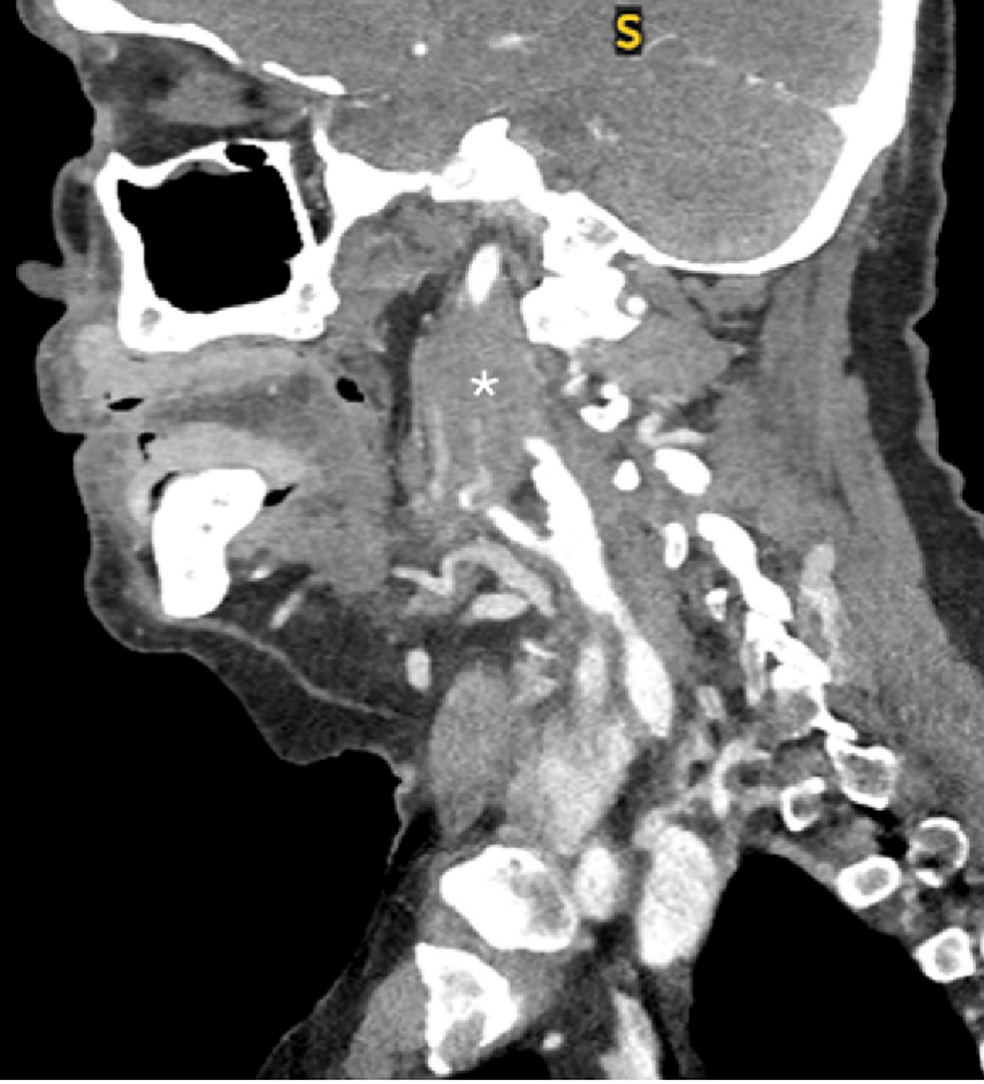
Sagittal plane: The bifurcation of the right common carotid is visualized before the parapharyngeal mass (asterisk) appears to encase the internal and external carotid arteries, obscuring them from view.

A 74-year-old male with a history of non-Hodgkin lymphoma was scheduled to undergo laryngoscopy with a biopsy of a large soft tissue mass in the right parapharyngeal space (Figures 1-3), as well as a large exophytic mass extending from the tongue base onto the right tonsil (Figure 2). These computerized tomography (CT) findings were consistent with lymphomatous involvement and associated metastatic right neck lymphadenopathy. He had previously been treated for large B-cell lymphoma with partial response to six cycles of ABVD and an autologous stem cell transplant.

**Figure 4:**
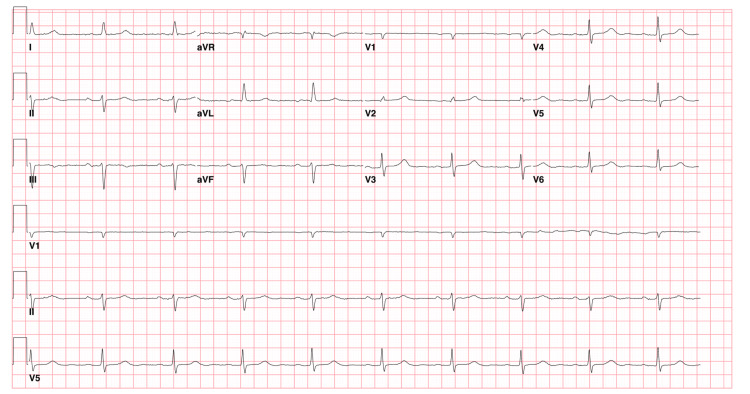
Electrocardiography (ECG): Sinus bradycardia at 58 beats per minute with the first-degree AV block (PR interval 220 ms), left-axis deviation, and low-voltage QRS.

During the preoperative period, the patient received 50 mcg of intravenous (IV) fentanyl for ongoing neck pain and then developed severe bradycardia, with heart rates (HRs) dropping to the 20s, and hypotension, with systolic blood pressure falling to the 70s. The procedure was postponed, and the patient was transferred to the intensive care unit (ICU) where his HR stabilized. Electrocardiography (ECG) showed sinus bradycardia with first-degree AV block with no evidence of ischemia or atrial fibrillation (Figure 4). He had no previous history of bradycardia. The patient denied any recent chest pain but had been experiencing mild stridor and shortness of breath attributed to the masses. The episode was hypothesized to stem from an augmented vagal reflex response in the context of tumor compression of the carotid sinus exaggerating the effect of fentanyl administration.

Due to his medical history of chronic obstructive pulmonary disease, tobacco use disorder, and coronary artery disease, the patient was transferred to a tertiary hospital for cardiac management before another attempt at the procedure. Cardiology evaluation, including echocardiography without abnormalities and an ejection fraction of 60%-65%, revealed no urgent issues and approved the patient for surgery, recommending avoiding the use of AV node-blocking agents.

The patient then underwent direct laryngoscopy with biopsy and tumor debulking without a recurrence of bradycardia and was discharged the next day. Throughout, his oxygen saturation remained high on a 3 L nasal cannula, and his hypertension was managed with a nicardipine drip instead of his usual beta-blocker. No opioids were used during the reattempt.

## Discussion

There are numerous considerations for the anesthesiologist and operating room team in cases of head and neck masses. In terms of induction, hemodynamic response to standard agents may be amplified by tumor proximity to the carotid sinus. This phenomenon is increasingly likely for tumors located in the parapharyngeal space and in cases of extensive cervical lymph node involvement, both of which features the patient displayed [1]. Fentanyl is frequently administered preoperatively for its utility in decreasing minimum alveolar concentration, alleviating pain, and blunting the hemodynamic sympathetic response to intubation [3]. However, this sympathetic blunting must be carefully considered for those patients at risk of reflex bradycardia, i.e., surgeries invoking a large vagal response or patients with concern for carotid compression. Beta-blockers, one of the patient’s scheduled medications, are one of the few anti-hypertensives not withheld on the day of surgery and may further dampen sympathetic activity. The patient had not yet received the totality of the scheduled induction agents, but one can imagine the effect of additional vasodilators, such as propofol and lidocaine. With so many contributors to hypotension, it’s reasonable in these presentations to consider alternative induction agents with stimulatory effects, such as ketamine [4]. Additionally, baseline hypertension should be controlled with agents such as dihydropyridine calcium channel blockers, rather than AV nodal blocking anti-hypertensives when there are concerns for bradycardia.

Intubation poses another concern in surgeries of head and neck masses, by way of mass effect or distortion of the glottis. In these patients, awake intubation is recommended with fiberoptic bronchoscopy and video laryngoscopy as the first line in the American Society of Anesthesiologists' difficult airway management guidelines [5]. If intubation remains unsuccessful, the algorithm advises invasive action with wire-guided fiberoptic retrograde intubation [4]. Neck hyperextension positioning is especially critical when preparing for a known difficult airway, such as this patient with stridor and shortness of breath at baseline. However, hyperextension of the neck can not only impede blood flow to the brain but also further compress the carotid, inciting the vagal reflex. Physicians must be hyperaware of patient positioning and possible sequelae, as hemodynamic depression of this origin may not respond to anticholinergics and may only be alleviated with repositioning of the head to a flexed position [6].

## Conclusions

Anesthesiologists and surgeons must exercise caution when intubating or operating on patients with head and neck masses. The proximity of a tumor to the carotid sinus can lead to compression, potentially causing hemodynamic collapse. In such cases, it is advisable to consider induction with stimulatory agents like ketamine, along with repositioning the patient's head if necessary. The use of sympatholytic drugs, including beta-blockers and opioids, should be carefully evaluated for their risks, and alternative options should be discussed thoroughly.
